# Quality of reporting in cardiac MRI, CT and SPECT diagnostic accuracy studies: Analysis of the impact of STARD criteria

**DOI:** 10.1186/1532-429X-15-S1-E76

**Published:** 2013-01-30

**Authors:** Edd N Maclean, Ian S Stone, Felix Ceelen, Xabier Garcia-Albeniz, Wieland H Sommer, Steffen E Petersen

**Affiliations:** 1 Advanced Cardiovascular Imaging, Barts and The London, London, UK; 2 Clinical Radiology, Grosshadern, Munich, Germany; 3 Epidemiology, Harvard School of Public Health, Boston, MA, USA

## Background

Diagnostic accuracy studies are essential for determining the clinical value of non-invasive cardiac imaging tests. Our aim was to assess the impact of the publication of the ‘Standards for reporting studies of diagnostic accuracy' (STARD) in 2003 on reporting quality.

## Methods

This paper reports on the reporting quality of cardiac computed tomography (CCT), single positron emission computed tomography (SPECT), and cardiac magnetic resonance (CMR) diagnostic accuracy studies in randomly selected groups of 50: ‘CMR 1995-2002', ‘CMR 2004-2011', ‘CCT f 1995-2002', and ‘CCT 2004-2011', ‘SPECT 1995-2002', ‘SPECT 2004-2011'. These 300 studies were read against the 25 STARD criteria (7500 items assessed) and their % adherence determined. Simple and multivariable linear regression models were developed.

## Results

The adherence to STARD criteria increased from 65.3% to 74.1% (p=0.0026) for CMR studies and from 61.6% to 79.0% (p<0.0001) for CCT studies following the introduction of STARD; The SPECT studies, however, did not show any change in adherence to STARD: 71.9% before and 71.5% after STARD (p=0.9223). Higher reporting quality is more associated with a journal's impact factor than with the journal's mentioning the STARD criteria in the authors' instructions. After adjustment for impact factor, the adherence to STARD criteria was similar in each calendar year before 2003 and since continues to increase by about (absolute) 1.5 %.

## Conclusions

The quality of reporting diagnostic accuracy studies in non-invasive cardiac imaging is satisfactory, but has improved since the introduction of STARD. The introduction of the STARD statement into the submission process, such as checklists, may further improve the quality of diagnostic accuracy studies.

## Funding

This work forms part of the research themes contributing to the translational research portfolio of Barts Cardiovascular Biomedical Research Unit which is supported and funded by the National Institute for Health Research.

